# Supplementary material for: HIVprotI: an integrated web based platform for prediction and design of HIV proteins inhibitors
Source: J Cheminform. 2018 Mar 9;10:12. doi: 10.1186/s13321-018-0266-y (PMC5845081; doi:10.1186/s13321-018-0266-y)
Supplement: Supplementary file 2 — Additional file 2. Source code of HIVProtI web server. [file 13321_2018_266_MOESM2_ESM.zip › HIVprotI_Source-code/hivprotI/jsme/JSME_parent_and_metabolites.html]

Your web browser must have JavaScript enabled
in order for this application to display correctly.

### Metabolism Database - Structure Input using JSME

### This is an example showing how to use JSME templates

|  |  |
| --- | --- |
| **Structure of Parent** | **Structure of Metabolite** |
|  |  |
|  | Add group (required anchor atom): D-Glucuronic acid (O) Sulfate (O) Phosphate (O) Glutathione (S) CoA (S) |

**How to add your own template**

Templates may be easily added using the JavaScript call `jsme.setTemplate('jme string')` where the jme string is the JME string of your template. The JME string may be generated for example here. The atom that should act as a connection point (root) of the template should by marked by :1 (for example the oxygen serving as root should have atomic symbol O:1).
  
For more information check also the HTML source of this page
